# Linearly Tunable Fano Resonance Modes in a Plasmonic Nanostructure with a Waveguide Loaded with Two Rectangular Cavities Coupled by a Circular Cavity

**DOI:** 10.3390/nano9050678

**Published:** 2019-05-01

**Authors:** Qiong Wang, Zhengbiao Ouyang, Yiling Sun, Mi Lin, Qiang Liu

**Affiliations:** 1 College of Physics and Optoelectronic Engineering, Shenzhen University, Shenzhen 518060, China; qwang@szu.edu.cn (Q.W.); sunyl@szu.edu.cn (Y.S.); linfengas111@szu.edu.cn (M.L.); qliu@szu.edu.cn (Q.L.); 2 THz Technical Research Center of Shenzhen University, Shenzhen University, Shenzhen 518060, China

**Keywords:** tunable Fano resonance modes, coupled-waveguide-cavity system, mode interference, surface plasmon polaritons, finite-element method

## Abstract

Linear tunability has important applications since it can be realized by using linear control voltage and can be used conveniently without requiring nonlinear scale. In this paper, a kind of plasmonic nanostructure with a waveguide loaded with two rectangular cavities coupled by a circular cavity is proposed to produce four Fano resonance modes. The transfer matrix theory is employed to analyze the coupled-waveguide-cavity system. By analyzing the property of each single cavity, it reveals that the Fano resonances are originated from the coupling effect of the narrow modes in the metal-core circular cavity and the broad modes in the rectangular cavities. Owing to the interference of different modes, Fano peaks have different sensitivities on the cavity parameters, which can provide important guidance for designing Fano-resonance structures. Furthermore, adjusting the orientation angle of the metal core in the circular cavity can easily tune the line profile of Fano resonance modes in the structure. Especially, the figure of merit (FoM) increases linearly with the orientation angle and has a maximum of 8056. The proposed plasmonic system has the advantage of high transmission, ultracompact configuration, and easy integration, which can be applied in biochemical detecting or sensing, ultra-fast switching, slow-light technologies, and so on.

## 1. Introduction

The collective electromagnetic oscillation due to the interaction of free electrons in a metal nanostructure with an electromagnetic wave, known as surface plasmon polariton (SPP), has been considered as one of the most promising effects for building highly integrated photonic circuits owing to its capabilities in overcoming diffraction limit of light wave, manipulating wave at a deep subwavelength scale, and producing extremely strong local field near the metal-dielectric surface [1,2,3,4,5,6]. By now, various kinds of nanoscale devices based on SPPs have been investigated theoretically and experimentally, such as optical filters, optoelectronic switches, optical amplifiers, and modulators [7,8,9]. Typically, plasmonic structures exhibiting conventional quasi-Lorentzian resonance are always characterized by symmetric and wide lineshape in their transmission spectra [10,11,12], which would limit their applications. To solve this problem, considerable attention has been paid to Fano resonances [13,14,15]. Fano resonance modes in SPP structures exhibit sharp and asymmetric spectral lineshape [16,17]. Due to the origination of Fano modes from interference, a small perturbation in background material may cause a rapid change in the operating state or the response of the structure. This property makes it appealing for detecting, sensing, switching, and slow-light technologies.

Recently, great efforts have been devoted to investigating multiple Fano resonances in plasmonic structures since they are very useful in applications that are simultaneously required at different frequencies [18,19,20,21]. For example, a kind of defective metallic disk that supports multiple Fano resonances has been proposed to realize plasmonic metamaterials, in which the multiple Fano resonances are resulted from a mutual coupling between the bright dipolar mode due to the wedge-shaped defect and dark multipole localized surface plasmon modes [22]. Also, a metal-dielectric Kretschmann structure has been reported to realize multiple Fano resonances formed by the coupling between the SPP mode and multi-order planar waveguide modes [23]. In addition, based on multiple Fano resonances, an ultracompact plasmonic sensor has been designed with a stub and a side-coupled split-ring resonator, and the Fano-typed lineshape exhibits a strong dependence on the symmetry breaking of the structure [24].

In all these approaches, the tunability of the device is neglected or difficult to realize. For applications, research on tunable multiple Fano resonances must be done extensively because they would bring great flexibility to plasmonic devices. Similar studies have emerged in acoustic wave transmission in a non-axisymmetric duct-cavity structure which consists of a cylindrical resonator and two cylindrical waveguides whose axes are shifted relative to the axis of the resonator by a distance. The research in this field needs to be extended to electromagnetic waves. [25] Especially for the linear tunability, it can be realized by using linear control voltage and can be used conveniently without requiring nonlinear scale.

In this paper, we propose an ultracompact plasmonic nanostructure to obtain linearly tunable multiple Fano resonance modes. It consists of a waveguide loaded with two rectangular cavities coupled with a metal-core circular cavity in metal-insulator-metal (MIM) structure, in which the metal-core circular cavity provides narrow modes, while the rectangular cavities provide wide modes, and the interaction between them, create four Fano resonance modes. The coupled-waveguide-cavity structure is analyzed by the transfer matrix theory. The different influence of cavity parameters on Fano resonance modes is also investigated. Furthermore, through adjusting the orientation angle of the metal core that greatly affects the coupling between the circular cavity and its two side-coupled rectangular cavities, the Fano resonance modes can be linearly tuned. The plasmonic structure may pave a new route to realize linearly tunable multiple Fano resonance modes, which can be widely applied in biochemical detecting or sensing, ultra-fast switching, slow-light technologies, and so on.

## 2. Structure and Theory Analysis

**Figure 1:**
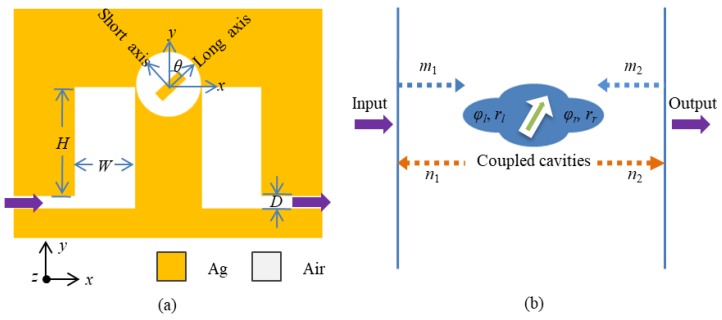
(**a**) The schematic diagram of a coupled-waveguide-cavity structure comprising a metal-core circular cavity, two side-coupled rectangular cavities, and MIM plasmonic waveguides at the input and output ports; and (**b**) its simplified transfer-matrix-theory model.

Figure 1a shows the schematic diagram of a two-dimensional plasmonic MIM nanostructure. The MIM-typed structure is chosen because it has the advantage of excellent wave confinement, applicable propagation length, and easy integration. In the structure, a circular cavity with a metal-strip core (abbreviated as a metal-core cavity in the following) is symmetrically located between two of the same rectangular cavities. The radius of the circular cavity is *R* = 180 nm. The width and the height of the rectangular cavities are set as *W* = 320 nm and *H* = 580 nm, respectively. Two waveguides are designed at the left and right sides to input and output electromagnetic waves, respectively. The width of the two waveguides is fixed as *D* = 70 nm so that only the fundamental mode can exist and propagate in the waveguide. The orange and white denote silver and air (*ε*_Air_ = 1), respectively. The frequency-dependent complex relative permittivity *ε*_Ag_ (*ω*) of silver is written as follows by the Drude model [26,27]:

(1) $$\varepsilon_{\mathrm{Ag}}(\omega )=\varepsilon_{\infty }-\frac{\omega_{p}^{2}}{\omega \;(\omega +i\gamma )},$$

where *ε*_∞_ is the dielectric constant at an infinite frequency, *γ* is the electron collision frequency, *ω* is the frequency of incident light and *ω_p_* is the bulk plasma frequency. The values of the parameters are *ε*_∞_ = 3.7, *ω*_p_ = 1.38 × 10^16^ Hz, and *γ* = 2.73 × 10^13^ Hz.

The input wave with the transverse magnetic field is launched at the left waveguide, and then transmits through the three coupled cavities, finally comes out from the right waveguide. In order to adjust the coupling of the circular cavity with the two rectangular cavities, the metal core in the circular cavity is rotated around the center point with its orientation defined by an angle *θ* between the y-axis and long axis of the metal strip. Meanwhile, to ensure effective adjustment, the length and width of the metal core are chosen as *M* = 180 nm and *N* = 70 nm, respectively. The total transmission of the system is defined as *P*_out_/*P*_in_, where *P*_in_ and *P*_out_ stand for the power flow obtained by integrating the Poynting vector over the cross-sections of the input and output ports, respectively.

In order to provide a comprehensive understanding of the proposed coupled-waveguide-cavity structure, the transmission is analyzed using the transfer matrix theory [28,29,30], as shown in Figure 1b. First, considering the coupled-waveguide-cavity structure without a metal-strip core in it, the scattering property at frequency *ω* can be described by a transfer matrix *T_s_* that relates the incoming and outgoing wave amplitudes *m*_1_ and *n*_1_ on the left side of the coupled-cavity structure, to the outgoing and incoming wave amplitudes *n*_2_ and *m*_2_ on the right side, as below:

(2) $$\left[\begin{array}{c} n_{2}\\ m_{2} \end{array}\right]=T_{s}\left[\begin{array}{c} m_{1}\\ n_{1} \end{array}\right]=\left[\begin{array}{cc} 1-\frac{i\eta }{\omega -\omega_{0}} & \frac{-i\eta }{\omega -\omega_{0}}\\ \frac{i\eta }{\omega -\omega_{0}} & 1+\frac{i\eta }{\omega -\omega_{0}} \end{array}\right]\left[\begin{array}{c} m_{1}\\ n_{1} \end{array}\right],$$

where *ω*_0_ and *η* are the center frequency and the width of resonance in the coupled-cavity structure without a metal-stripe core, respectively. When adding a metal core in the circular cavity, it can be regarded as an important element that affects the phases and reflections of the left and right waves. The transfer matrix *T_s_*′ for the entire waveguide-cavity system can be modified to be:

(3) $$T_{s}{}^{\prime }=T_{left}T_{s}T_{right}=\frac{-i}{\sqrt{1-r_{l}{}^{2}}}\left[\begin{array}{cc} -1 & -r_{l}\\ r_{l} & 1 \end{array}\right]\left[\begin{array}{cc} e^{i\varphi_{l}} & 0\\ 0 & e^{-i\varphi_{l}} \end{array}\right]T_{s}\frac{-i}{\sqrt{1-r_{r}{}^{2}}}\left[\begin{array}{cc} e^{i\varphi_{r}} & 0\\ 0 & e^{-i\varphi_{r}} \end{array}\right]\left[\begin{array}{cc} -1 & -r_{r}\\ r_{r} & 1 \end{array}\right],$$

where (*φ_l_*, *r_l_*) and (*φ_r_*, *r_r_*) denote the phase and reflection changes of the left and right waves induced by the added metal-core. In the structure, the left and right straight waveguides can be considered as in states 0› and 0′›, respectively. The left rectangular cavity, the metal-core cavity, and the right rectangular cavity can be considered as in states 1›, 2› and 1′›, respectively. When the resonance mode is formed in the coupled cavities, the process can be considered as 0›→1›→2›→1′›→0′›. When *θ* is changed, the field pattern in the circular cavity or the rectangular cavities is adjusted, then the parameters (*φ_l_*, *r_l_*) for the coupling 1›→2› and (*φ_r_*, *r_r_*) for the coupling 2›→1′› are affected. From Equation (3), the transmission *T*_all_ (*ω*) can be written out as:

(4) $$\begin{array}{l} T_{all}(\omega )={\left|\frac{1}{T_{s,22}^{\prime }}\right|}^{2}\\ ={\left|\frac{\;-\sqrt{\left(1-r_{l}^{2})(1-r_{r}^{2}\right)}(\omega -\omega_{0})}{\left[e^{-i(\varphi_{l}+\varphi_{r})}-r_{l}r_{r}e^{i(\varphi_{l}+\varphi_{r})}\right](\omega -\omega_{0})+i\eta \left[r_{l}r_{r}e^{i(\varphi_{l}+\varphi_{r})}+e^{-i(\varphi_{l}+\varphi_{r})}-(r_{r}e^{-i(\varphi_{l}-\varphi_{r})}+r_{l}e^{i(\varphi_{l}-\varphi_{r})})\right]}\right|}^{2} \end{array}$$

Furthermore, when the symmetry of the structure is considered, we have *φ_l_* = *φ_r_* and *r_l_* = *r_r_*. Two new parameters are defined as *φ = φ_l_* = *φ_r_* and *r = r_l_* = *r_r_*. We think that *φ* and *r* depend not only on *θ*, but also on the wavelength. This can be explained as follows. If *θ* is fixed, the field pattern in the circular cavity or the rectangular cavities is changed for different wavelength, which results in the corresponding change in the phase and reflection of the left and right waves induced by the added metal-core. The transmission *T*_all_ (*ω*) in Equation (4) can be changed as:

(5) $$T_{all}(\omega )={\left|\frac{\;(r^{2}-1)(\omega -\omega_{0})}{\left(e^{-2i\varphi }-r^{2}e^{2i\varphi })(\omega -\omega_{0})+i\eta (r^{2}e^{2i\varphi }+e^{-2i\varphi }-2r\right)}\right|}^{2},$$

It can be seen from Equation (5) that when the orientation angle *θ* of the metal-stripe core is changed, the parameters *φ* and *r* vary, resulting in variation in the coupling of metal-core circular cavity with the two rectangular cavities. Therefore, a complex change takes place in transmission *T*_all_(*ω*), and Fano resonances can be formed and tuned when *θ* is changed.

## 3. Analysis and Discussions

### 3.1. The Mechanism of the Multiple Fano Resonances in the Coupled-Cavity Structure

Based on the theoretical analysis presented above, the optical response of the designed structure is numerically investigated by using the finite-element method (FEM) with comsol multiphysics. The whole system is divided into about 6 × 10^4^ cell grids and the convergence test is done in the process of mesh generation to ensure the accuracy of the calculation. The calculated domain is surrounded by perfectly matched layers to absorb the electromagnetic wave going out of the structure.

**Figure 2:**
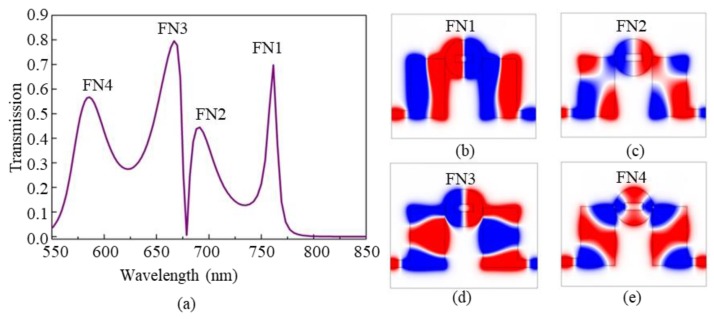
(**a**) The transmission of the coupled-waveguide-cavity structure, and the *H_z_* magnetic-field distributions at the Fano peaks of (**b**) FN1 with *λ* = 761 nm, (**c**) FN2 with *λ* = 690 nm, (**d**) FN3 with *λ* = 668 nm, and (**e**) FN4 with *λ* = 585 nm.

Figure 2a shows the calculated transmission spectrum of the coupled-waveguide-cavity structure. We observe that four resonance peaks with high transmissions form at the wavelengths of *λ* = 761 nm, λ = 690 nm, λ = 668 nm, and *λ* = 585 nm, respectively. They exhibit sharp and asymmetric Fano-type line shapes. For convenience, the four Fano peaks are indicated by FN1, FN2, FN3, and FN4, respectively. As we know, Fano resonances usually are resulted from the coupling of narrow discrete states and wide continuous states. Furthermore, the magnetic-field *H_z_* distributions at the peaks are shown in Figure 2b–e. It is obvious that they result from the coupling of different cavity modes. In order to explore the mechanism of the Fano resonances in detail, it is necessary to separately study the two kinds of basic cavities, i.e., the metal-core cavity and the rectangular cavity.

**Figure 3:**
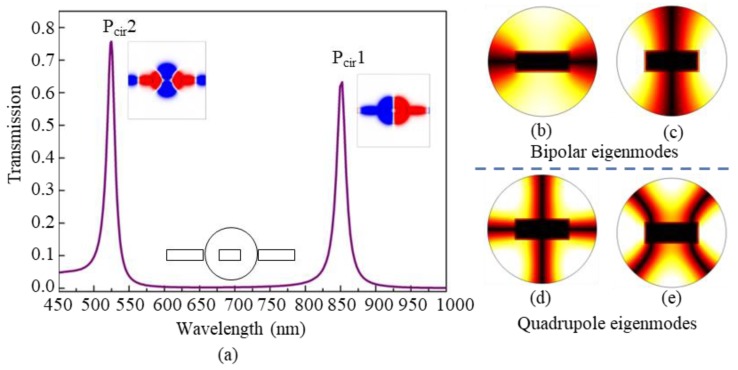
(**a**) The transmission of the metal-core circular cavity coupled with a waveguide, where the black-line inset shows the detailed structure, and the color insets show the magnetic-field *H_z_* distributions at the transmission peaks of P_cir_1 (*λ* = 852 nm) and P_cir_2 (*λ* = 526 nm); (**b**,**c**) the |*H*| distributions of dipole eigenmodes, and (**d**,**e**) the |*H*| distributions of quadrupole eigenmodes, for the metal-core circular cavity without coupling with a waveguide.

Figure 3a shows the calculated transmission spectrum of a metal-core cavity only coupled with a waveguide. The detailed structure is illustrated in the black-line inset in the middle near the wavelength scale. The radius *R* of the cavity is 180 nm. The width *M*, length *N*, and orientation angle *θ* of the metal core are 180 nm, 70 nm, and 90^o^, respectively. As expected, there are two high and narrow transmission peaks at the wavelengths of *λ* = 852 nm (P_cir_1 mode) and *λ* = 526 nm (P_cir_2 mode), respectively. The *H_z_* magnetic-field distributions at the resonance peaks are shown by the color insets. They correspond to a dipole mode and a quadrupole mode, which are anti-symmetric and symmetric about the y-axis (vertical axis), respectively. Furthermore, the eigenmodes of the metal-core circular cavity without a waveguide are simulated and shown in Figure 3b–e, which can be divided into two kinds of basic modes, i.e., bipolar modes shown in Figure 3b,c, and quadrupole modes shown in Figure 3d,e. It can be concluded that P_cir_1 and P_cir_2 are resulted from the coupling effect of SPP waveguide modes and the eigenmodes from the metal-core cavity.

**Figure 4:**
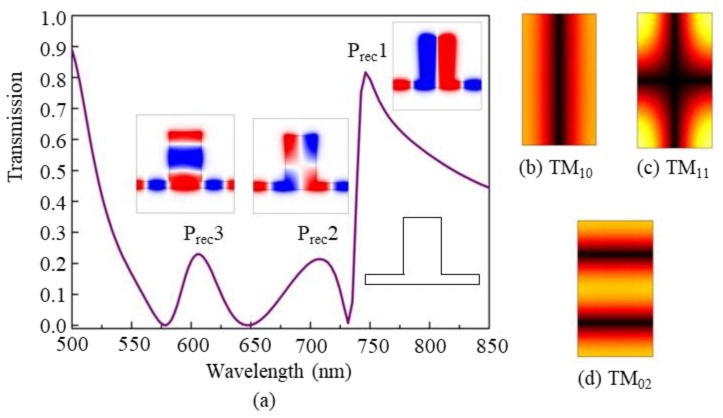
(**a**) The transmission of the rectangular cavity coupled with a waveguide, where the black-line inset indicates the detailed structure, and the color insets show the simulated magnetic-field *H_z_* distributions at the transmission peaks of modes P_rec_1 (*λ* = 746 nm), P_rec_2 (*λ* = 709 nm), and P_rec_3 (*λ* = 606 nm). (**b**) The |*H*| distributions of TM_10_ mode, (**c**) of TM_11_ mode, and (**d**) of TM_02_ mode, in the rectangular cavity not coupled with a waveguide.

Figure 4a shows the calculated transmission spectrum of a rectangular cavity only coupled with a waveguide. The detailed structure is illustrated in the black-line inset at the right side near the wavelength scale. The width *W* and length *H* of the cavity are 320 nm and 580 nm, respectively. We can observe that three resonance peaks are generated at the wavelengths of *λ* = 746 nm (P_rec_1 mode), *λ* = 709 nm (P_rec_2 mode) and *λ* = 606 nm (P_rec_3 mode), respectively. Different from the case of the metal-core cavity discussed above, the three peaks are relatively wide. This is because the rectangular cavity is chosen to be quite large. The magnetic-field *H_z_* distributions at the resonance peaks are displayed by the color insets. We can see that they exhibit different resonance patterns. According to the mode patterns, the mode in the rectangular cavity can be regarded as TM*_mn_*, where *m* and *n* are the mode numbers (equal to the mode order numbers) in the horizontal and vertical directions, respectively. So, the modes P_rec_1, P_rec_2, and P_rec_3 can be also regarded as TM_10_ mode, TM_11_ mode, and TM_02_ mode, respectively, which means that the mode P_rec_1 is the 1st-order resonance in the horizontal direction, the mode P_rec_2 is the 1st-order resonance in both horizontal and vertical directions, and the mode P_rec_3 is the 2nd-order resonance along the vertical direction. Furthermore, the eigenmodes of the rectangular cavity without a waveguide are shown in Figure 4b–d, which can be regarded as TM_10_ mode, TM_11_ mode, and TM_02_ mode, respectively. It can be concluded that the modes P_rec_1, P_rec_2, and P_rec_3 are resulted from the coupling effect of the SPP waveguide modes and the eigenmodes from the rectangular cavity.

Compared the *H_z_* patterns in Figure 2, Figure 3 and Figure 4, it can be inferred that the four Fano resonances in the coupled-cavity structure originate from the interference of different modes from the metal-core cavity and the rectangular cavity, i.e., the Fano peaks FN1, FN2, and FN3 are formed by the coupling effects of the modes P_cir_1 and P_rec_1, P_cir_1 and P_rec_2, and P_cir_1 and P_rec_3, respectively. Compared to the three cases shown above, FN4 is relatively complicated, which is formed by the coupling effect of the modes P_cir_2, P_rec_2, and P_rec_3.

### 3.2. The Sensitivity of Multiple Fano Resonances under Different Cavity Parameters

**Figure 5:**
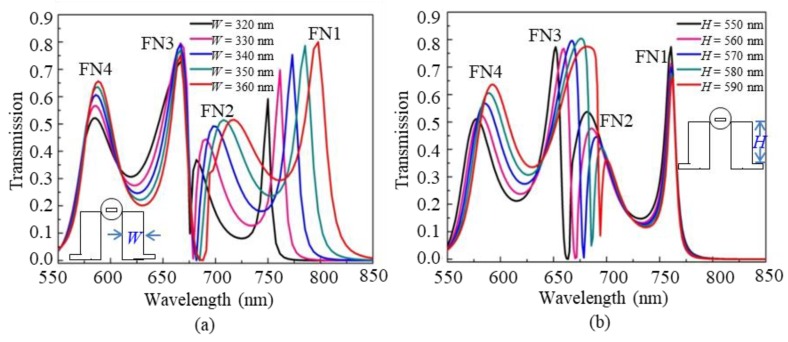
Transmission changes for (**a**) different width *W* of the rectangular cavity, and (**b**) different height *H* of the rectangular cavity.

In order to further investigate the properties of the coupled-waveguide-cavity structure, the influence of the cavity parameters on the Fano peaks FN1, FN2, FN3, and FN4 is analyzed. Figure 5a depicts the change in the transmission of the coupled-waveguide-cavity structure when the width of the rectangular cavity increases from *W* = 320 nm to *W* = 360 nm (keeping *H* = 580 nm). The result shows that FN1, FN2, and FN4 have obvious redshifts Δ*λ* with increasing values of 46 nm, 36 nm, and 5 nm, respectively, while FN3 nearly has no change. Furthermore, Figure 5b shows the change when the height of the rectangular cavity increases from *H* = 550 nm to *H* = 590 nm (keeping *W* = 320 nm). It shows that FN2, FN3, FN4 have obvious redshifts Δ*λ* with increasing values of 16 nm, 30 nm, and 20 nm, respectively, while FN1 nearly has no change.

This phenomenon can be explained from the resonance theory of the rectangular cavity. For FN1 in coupled-waveguide-cavity structure (see Figure 2b), the field pattern in the rectangular cavity is TM_10_ mode, which means that the resonance is sensitive to the change of cavity parameter in horizontal direction and insensitive to that in vertical direction, thus FN1 is not affected by the change of the height *H* of the rectangle cavity as shown in Figure 5b. On the other hand, for FN3 in the coupled-waveguide-cavity structure (see Figure 2d), the field pattern of the rectangular cavity is TM_02_, which means that the resonance is sensitive to the change of cavity parameter in the vertical direction and insensitive to that in the horizontal direction, thus FN3 is not affected by the change of the width *W* of the rectangle cavity, as shown in Figure 5a. For FN2 and FN4, they are greatly affected by the parameters *H* and *W* since both of their rectangular cavities contain TM_11_ mode (see Figure 2c,e) that are sensitive to the vertical and horizontal parameters. According to this characteristic, it is easy to tune the multiple Fano resonances by choosing appropriate structure parameters.

### 3.3. Realizing Linearly Tunable Fano Resonances by Adjusting the Coupling of Cavities

**Figure 6:**
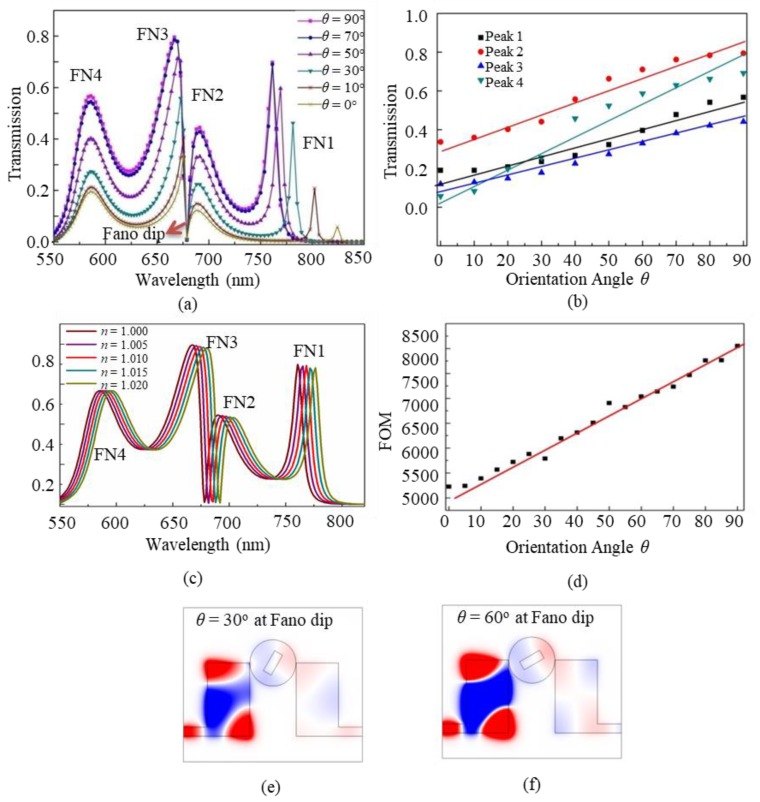
The influence of the orientation angle *θ* of the metal core on the transmission spectra (**a**), the aptitudes of the four Fano peaks (**b**), and the transmission related to the change of the refractive index at *θ* = 30° (**c**), and the FoM values for different *θ* (**d**), the *H_z_* distributions at the Fano dips for *θ* = 30° (**e**) and *θ* = 60° (**f**).

Next, we will investigate the tunability of Fano resonances by adjusting the orientation angle *θ* of the metal core. When the angle *θ* is changed from 0° to 90°, the transmission of the coupled-cavity structure is displayed in Figure 6a. It shows that the transmission is enhanced with the increase of *θ*. For the four Fano peaks, their amplitudes increase linearly with *θ*, as shown in Figure 6b. When *θ* = 90°, the aptitudes reach the maximum values of 0.69, 0.44, 0.79, and 0.56, respectively. The reason for this phenomenon is as follows. When the long axis of the metal core is adjusted along the y-axis (*θ* = 0°), it can be considered that the metal core plays a role of light blocking, resulting in low transmission. This case can be regarded as an “off” state. When the long axis of the metal core is adjusted along the x-axis (*θ* = 90°), it can be regarded as an excellent coupling bridge that efficiently connects the waves among the cavities, leading to high transmission. This case can be regarded as an “on” state. Beside the on-off switch application, it is easily known that this structure can also be used as an adjustable attenuator.

As can be seen from the above results, Fano modes exist in the structure. Since Fano modes are sensitive to background refractive index, so the proposed structure can also be used as refractive index sensors. Now we investigate the sensing performance of the structure by calculating the figure of merit (FoM), which describes the relative transmission variation at a fixed wavelength *λ* induced by the change of refractive index, and is an important parameter reflecting the sensitivity of Fano resonance to the background dielectric material. We define FoM* = [*T* (*λ,n*_0_ *+* Δ*n*) *− T* (*λ,n*_0_)]/(Δ*n·T* (*λ,n*_0_)), and FoM = max [FoM*], where *T* (*λ,n*_0_), *T* (*λ,n*_0_ *+* Δ*n*) are the transmissions at wavelength *λ* for the coupled structure with the refractive indices *n*_0_ and *n*_0_ *+* Δ*n* of the background dielectric material, respectively [31]. The transmission generally has a redshift with the increase of the background dielectric material, as shown in the example of *θ =* 90° in Figure 6c. Furthermore, the FoM value is calculated when *θ* is scanned from *θ =* 0° to *θ =* 90°, as shown in Figure 6d. We can find that the FoM value is highest at λ = 678 nm, exactly near the Fano dip. Especially, the FoM value increases linearly with *θ*, and it reaches the maximum value 8056 at *θ =* 90°. This is understandable because when *θ* increases, the transmission increases, so the slop of the Fano mode increases also, and as a result, the Fano mode will be more sensitive to the change of the background refractive index. The high FoM contributes to the sharp Fano lineshape in transmission and high sensitivity to the change in background refractive index. The characteristic of linear tunability has important applications since it can be realized by using linear control voltage and can be used conveniently without requiring nonlinear scale.

Another interesting result in Figure 6a is that the Fano dip at λ = 678 nm nearly has no change with the increase of *θ.* In order to understand this phenomenon, the *H_z_* distributions of Fano dip are investigated. We choose *θ* = 30° and *θ* = 60° as two examples, as shown in Figure 6e,f. It is obvious that the strong field distribution only exists in the left rectangular cavity, while in the right rectangular cavity and the metal-core circular cavity it is very weak. This is because at the Fano dip, the transmission is very low so that almost no energy goes to the right rectangle cavity. It then can be inferred that when *θ* changes, it has little effect on the field pattern. So the Fano dip is insensitive to the change of *θ*. In addition, the highest peak-valley ratio is close to 0.795/0.00411 = 193. The steep change in spectral line can be found considerable applications in nanosensors, filters, switches, modulators, and nonlinear devices. Moreover, the plasmonic structure has the advantage of high transmission, ultracompact configuration, and easy fabrication, which is suitable for integrating with other devices.

The proposed structure has wide applications in optical integrated on-chip devices since the MIM-typed configuration exhibits the advantages in convenient fabrication and easy integration. Moreover, the dimensions to achieve different resonances described in the paper are span about 80 nm, which is within the capability of today’s technologies. For the experiment, the patterns can be fabricated by lithography with the lift-off process. The silver film can be deposited by a magnetron sputtering technique. For mass production, the nanoimprint lithography is a method of fabricating nanometer-scale patterns, which is low in cost and high in resolution.

## 4. Conclusions

In summary, we have numerically demonstrated linearly tunable Fano resonances in a plasmonic MIM nanostructure, in which a metal-core circular cavity is side-coupled with two rectangular cavities. Four Fano resonances are formed, which originate from the coupling effect of the narrow modes from the metal-core circular cavity and the broad modes from the rectangular cavities. Owing to the interference of different modes, Fano resonances have different sensitivities under different cavity parameters. Through adjusting the orientation angle of the metal core, the Fano resonance sensitivity and amplitude can be linearly tuned. The FoM value can reach as high as 8056. The proposed ultracompact nanostructure holds potential applications in biochemical detecting or sensing, lasing, switching, and slow-light technologies.

## References

[1] Caldwell J.D., Lindsay L., Giannini V., Vurgaftman I., Reinecke T.L., Maier S.A., Glembocki O.J. (2015). Low-loss, infrared and terahertz nanophotonics using surface phonon polaritons. Nanophotonics.

[2] Lin J., Balthasar Mueller J.P., Wang Q., Yuan G.H., Antoniou N., Yuan X.C., Capasso F. (2013). Polarization-controlled tunable directional coupling of surface plasmon polaritons. Science.

[3] Zhang F., Zhang Y.J., Zhang Y.J., Chen L., Liu Y., Yang J.H. (2018). Ag nanotwin-assisted grain growth-induced by stress in SiO_2_/Ag/SiO_2_ nanocap arrays. Nanomaterials.

[4] Williams C.R., Andrews S.R., Maier S.A., Fernandez-Dominguez A.I., Martin-Moreno L., Garcia-Vidal F.J. (2008). Highly confined guiding of terahertz surface plasmon polaritons on structured metal surfaces. Nat. Photonics.

[5] Zayats A.V., Smolyaninov I.I., Maradudin A.A. (2005). Nano-optics of surface plasmon polaritons. Phys. Rep..

[6] Darweesh A.A., Bauman S.J., Debu D.T., Herzog J.B. (2018). The role of rayleigh-wood anomalies and surface plasmons in optical enhancement for nano-gratings. Nanomaterials.

[7] Berini P., Leon I.D. (2012). Surface plasmon-polariton amplifiers and lasers. Nat. Photonics.

[8] Melikyan A., Lindenmann N., Walheim S., Leufke P.M., Ulrich S., Ye J., Vincze P., Hahn H., Schimmel T., Koos C. (2011). Surface plasmon polariton absorption modulator. Opt. Express.

[9] Vinnakota R.K., Genov D.A. (2014). Terahertz optoelectronics with surface plasmon polariton diode. Sci. Rep..

[10] Lu F., Li G.Y., Li K., Wang Z.H., Xu A.S. (2012). A compact wavelength demultiplexing structure based on arrayed MIM plasmonic nano-disk cavities. Opt. Commun..

[11] Zhang Z., Shi F.H., Chen Y.H. (2015). Tunable multichannel plasmonic filter based on coupling-induced mode splitting. Plasmonics.

[12] Wang T.B., Wen X.W., Yin C.P., Wang H.Z. (2009). The transmission characteristics of surface plasmon polaritons in ring resonator. Opt. Express.

[13] Amin M., Ramzan R., Siddiqui O. (2017). Fano resonance based ultrahigh-contrast electromagnetic switch. Appl. Phys. Lett..

[14] Li A., Bogaerts W. (2017). An actively controlled silicon ring resonator with a fully tunable Fano resonance. APL Photonics.

[15] Chenari Z., Latifi H., Ranjbar-Naeini O.R., Zibaii M.I., Behroodi E., Asadollahi A. (2018). Tunable Fano-like lineshape in an adiabatic tapered fiber coupled to a hollow bottle microresonator. J. Light. Technol..

[16] Khan A.D., Amin M. (2017). Polarization selective multiple Fano resonances in coupled T-shaped metasurface. IEEE Photonics Technol. Lett..

[17] Wang M.S., Krasnok A., Zhang T.Y., Scarabelli L., Liu H., Wu Z.L., Liz-Marzan L.M., Terrones M., Alu A., Zheng Y.B. (2018). Tunable Fano resonance and plasmon-exciton coupling in single Au nanotriangles on monolayer WS_2_ at room temperature. Adv. Mater..

[18] Li W.Y., Su Y., Zhai X., Shang X.J., Xia S.X., Wang L.L. (2018). High-Q multiple Fano resonances sensor in single dark mode metamaterial waveguide structure. IEEE Photonics Technol. Lett..

[19] Khan A.D. (2014). Multiple Fano resonances in bimetallic layered nanostructures. Int. Nano Lett..

[20] Muhammad N., Khan A.D., Deng Z.L., Khan K., Yadav A., Liu Q., Ouyang Z.B. (2017). Plasmonic spectral splitting in ring/rod metasurface. Nanomaterials.

[21] Zhang Y.B., Liu W.W., Li Z.C., Li Z., Cheng H., Chen S.Q., Tian J.G. (2018). High-quality-factor multiple Fano resonances for refractive index sensing. Opt. Lett..

[22] Chen L., Xu N.N., Singh L., Cui T.J., Singh R.J., Zhu Y.M., Zhang W.L. (2017). Defect-induced Fano resonances in corrugated plasmonic metamaterials. Adv. Opt. Mater..

[23] Yang L., Wang J.C., Yang L.Z., Hu Z.D., Wu X.J., Zheng G.G. (2018). Characteristics of multiple Fano resonances in waveguide-coupled surface plasmon resonance sensors based on waveguide theory. Sci. Rep..

[24] Ren X.B., Ren K., Cai Y.X. (2017). Tunable compact nanosensor based on Fano resonance in a plasmonic waveguide system. Appl. Opt..

[25] Sadreev A., Pilipchuk A.S., Pilipchuk A.A. (2018). Tuning of Fano Resonances by Waveguide Rotation. Fano Resonances in Optics and Microwaves.

[26] Chen Z., Song X.K., Duan G.Y., Wang L.L., Yu L. (2015). Multiple Fano resonances control in MIM side-coupled cavities systems. IEEE Photonics J..

[27] Zhang B.H., Wang L.L., Li H.J., Zhai X., Xia S.X. (2016). Two kinds of double Fano resonances induced by an asymmetric MIM waveguide structure. J. Opt..

[28] Chen J.J., Li Z., Lei M., Fu X.L., Xiao J.H., Gong Q.H. (2012). Plasmonic Y-splitters of high wavelength resolution based on strongly coupled-resonator effects. Plasmonics.

[29] Fan S.H. (2002). Sharp asymmetric line shapes in side-coupled waveguide-cavity systems. Appl. Phys. Lett..

[30] Xu Y., Li Y., Lee R.K., Yariv A. (2000). Scattering-theory analysis of waveguide-resonator coupling. Phys. Rev. E.

[31] Wang Y.L., Li S.L., Zhang Y.Y., Yu L. (2018). Independently formed multiple Fano resonances for ultra-high sensitivity plasmonic nanosensor. Plasmonics.

